# Bundled Interventions to Improve CHG Bathing Compliance as a Strategy to Decrease MRSA Healthcare-Associated Infections

**DOI:** 10.1017/ash.2025.305

**Published:** 2025-09-24

**Authors:** Vanessa Kung, Lynette Hathaway, Carli Viola-Luqa, Joseph Penzelik, Shelia Cloud-Woods, Mary Jo Zubaty, Adrian Clifford, Jody Feigel, Elise Martin

**Affiliations:** 1 Pittsburgh VA Hospital; 2 VA Pittsburgh Healthcare System; 3 Pittsburgh VA Health System; 4 VAPHS; 5 VA Pittsburgh; 6 The US Department of Veterans Affairs; 7 Jody Feigel, VAPHS; 8 VA Pittsburgh Healthcare System

## Abstract

**Background:** Universal decolonization using chlorhexidine gluconate (CHG) foaming soap in a hospital system has been shown to reduce healthcare-associated infections (HAIs) and colonization by multidrug-resistant organisms. Limited data exist on optimal strategies to improve compliance, and the impact of improved compliance on HAI rates. This study evaluates the effect of increasing CHG compliance on MRSA HAI rates. **Methods:** In 2022, our acute care VA hospital started universal CHG bathing treatment, by requiring a daily CHG bath for all patients in intensive care units and medical/surgical floors, unless contraindicated. Despite this, compliance was below goal. We performed root cause analyses to identify factors contributing to poor compliance, and then initiated a bundled intervention, including nursing staff education on the benefits of CHG bathing to reduce HAIs, how to reframe discussions with patients about refusals, removal of one alternative soap product from the inventory, and moving the CHG bathing product in clean supply rooms to be in proximity with other patient hygiene products for easier access. We evaluated the utilization of CHG bathing products through inventory data on utilization of 4 fluid ounce bottles of 4.0% weight/volume CHG solution, documentation of at least one CHG bath in the electronic medical record (EMR) per unique hospitalization, and HAI rates per National Healthcare Safety Network (NHSN) definitions for methicillin resistant Staphylococcus aureus (MRSA), before (08/2023-02/2024) and after (03/2024-12/2024) implementation of the bundle. **Results:** Identified barriers to CHG adherence included use of less effective alternative soap agents, perceptions of patient skin irritation from CHG, difficulty integrating CHG into existing workflows, and lack of understanding of the benefits of CHG bathing. After bundled interventions, inventory usage CHG bottles increased from 170 to 270 bottles per 1,000 bed days of care (BDOC) (p **Conclusion:** An intervention of staff education, removal of an alternative soap product, and improving access to CHG bathing products in supply rooms, resulted in improved CHG bathing adherence, and was associated with a reduction in MRSA HAIs in an acute care VA hospital. Interestingly, the decrease in MRSA HAIs was achieved despite an absence of complete adherence. Further data on additional strategies to improve compliance and strategies to improve healthcare worker documentation should be explored.

